# Case Report: A *de novo* CTNNB1 Nonsense Mutation Associated With Neurodevelopmental Disorder, Retinal Detachment, Polydactyly

**DOI:** 10.3389/fped.2020.575673

**Published:** 2020-12-11

**Authors:** Zhongling KE, Yanhui CHEN

**Affiliations:** Department of Pediatrics, Fujian Medical University Union Hospital, Fuzhou, China

**Keywords:** CTNNB1 gene, neurodevolpmental disorder, retinal detachment, polydactyly, case report

## Abstract

*CTNNB1* gene mutation was firstly reported related to intellectual disability in 2012, to explore the clinical phenotype and genotype characteristics of *CTNNB1* mutation, we collected and analyzed the clinical data of a child with a neurodevelopmental disorder caused by a mutation of *CTNNB1*. The child had dysmorphic features, microcephaly, hypotonia, polydactyly, retinal detachment, and neurodevelopmental disorder, with a *de novo* mutation of *CTNNB1* c.1603C > T, p.R535X. The patient was diagnosed as Neurodevelopmental disorder with spastic diplegia and visual defects (NEDSDV) and was given rehabilitation training. After 4 months of rehabilitation training, she improved in gross motor function. We found that *CTNNB1* mutation can cause neurodevelopmental disorder, which could be accompanied by retinal detachment and polydactyly. The retinal detachment had only been reported in two Asian patients, and we firstly reported the phenotype of polydactyly in the *CTNNB1* mutation. This report not only helps to expand the clinical phenotype spectrum of the *CTNNB1* gene mutation but also prompts a new insight into genetic diagnosis in patients with a neurodevelopmental disorder, retinal detachment, and polydactyly.

## Introduction

*CTNNB1* (OMIM: 116806) gene encodes β–catenin protein, which is an integral part of the cadherin/catenin complex and is related to the activation of the Wnt signaling pathway.

It found that β–Catenin knockout mice showed the same behavior as autism spectrum disorder (1), while the loss of function of the *CTNNB1* gene was related to intelligence disorder (2). *CTNNB1* gene mutations have a variety of phenotypes, such as Colorectal cancer, Exudative vitreoretinopathy 7, Hepatocellular carcinoma, Medulloblastoma, Neurodevelopmental disorder with spastic diplegia and visual defects and Pilomatricoma (3). Neurodevelopmental disorder with spastic diplegia and visual defects(NEDSDV, OMIM:615075) was the only one presenting neurological impairment, which was characterized by global developmental delay, impaired intellectual development, axial hypotonia, and dysmorphic craniofacial features with microcephaly. Many patients have visual abnormalities, ranging from strabismus to optic nerve atrophy and retinal abnormalities. Spasticity may also occur in the affected individuals, particularly of the lower extremities, and may have behavioral abnormalities (4).

At present, 33 cases of the disease have been reported in the world (2, 5–11), with only two in Asia (8, 10). This paper reports the clinical characteristics and genetic analysis of the third case of neurodevelopmental disorder caused by *CTNNB1* gene mutation in the Asian population, and we found that polydactyly may be a new feature of *CTNNB1* mutation.

## Case Report

The patient was a 15-month-old girl who came to our department developmental retardation. She is the second child of Chinese parents who are both healthy, young, non-consanguineous. She has a healthy elder brother. During pregnancy, ultrasound scan showed intrauterine growth retardation. She weighed 2.35 kg at birth. The family history was negative for birth defects, developmental delay, intellectual delay, and/or any other neurological disorders.

**Figure 1:**
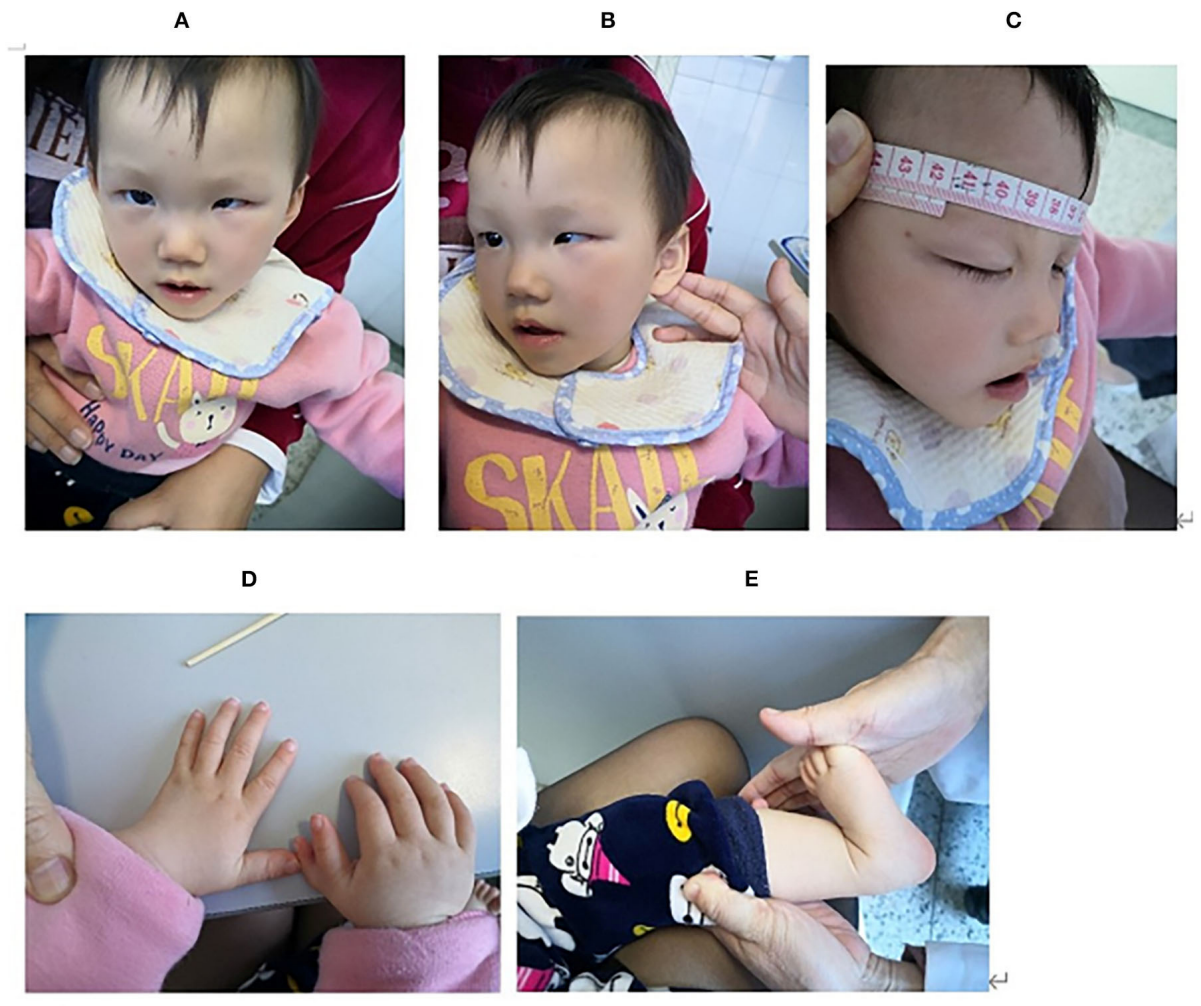
**(A,B)** Craniofacial dysmorphism; **(C)** microcephaly; **(D)** polydactyly; **(E)** Hypotonia.

After a normal neonatal period, she was found with motor retardation when she can't raise her head at 3 months old. At the age of 4 months, she had an ophthalmologic examination and was found blindness in the left eye when her parents wanted to treat strabismus her left eye. Ultrasonic examination showed a retinal detachment in the left eye. Fortunately, her right eye was normal. The patient was evaluated in our department at 15 months. She can raise her head but unstably, and still can't sit. The language barrier is very serious. She can make some sounds, but she can't speak. er parents found her intelligence lower than her peers. Physical examination showed as follows: weight 7.5 kg (−2.46 SD), height 72.5 cm(−2.29 SD), occipitofrontal circumference(OFC) 41 cm (−4.17 SD), anterior frontal was closure, light hair color, fair skin, low set ears, flat nasal bridge, strabismus in the left eye, thin upper lip, polydactyly in the right hand, but the foot does not have polydactylous. The systolic murmur of grade 2/6 can be heard in the 3–4 intercostals of the precordial area. The lung and abdomen are normal. The tension of trunk and peripheral limbs decreased significantly. Bilateral tendon reflexes were weakened, Babbitt's sign was negative, and no sign of spastic paralysis (Figures 1A–E). Auxiliary examination: the whole blood count, urine routine test, biochemical test, thyroid function, and the level of 25 (OH) D3 were all normal. Color Doppler echocardiography showed atrial septal defect, right heart enlargement, pulmonary hypertension, and tricuspid regurgitation (Table 1).

**Table 1:** Clinical findings in our patient and patients reported (4) before with the same mutation.

	**Our patient**	**Patient 1**	**Patient 2**
**Gender**	**Female**	**Male**	**Male**
Gestational weeks	38	40	41
Birth weight (kg)	2.35	3.05	3.4
Maternal age	24	39	28
Paternal age	26	43	38
Age of onset of symptoms	Birth	3 months	Birth
Age at diagnosis	1 year 3 months	3 years 3 months	14 years
Prenatal issues	Intrauterine growth retardation	None	None
Neonatal issues	None	Low oxygen saturation	Persistent pulmonary hypertension
Growth Parameters (SD)	At 15months: OFC 41 cm (4.17SD) Weight 7.5 kg (2.46 SD) Height 72.5 cm (2.29 SD)	At 21 months: OFC 43 cm (5.25 SD) Weight 10.45 kg (1.29 SD)Height 83.5 cm (0.31 SD)	At 6 years: OFC50.4 cm (1.93 SD) Weight 26 kg (1.17 SD)
Microcephaly	Yes	Yes	No
Fair skin (ethnic origin)	Yes(Chinese)	Yes (Scottish Caucasian)	Yes (White British)
Hair	Light hair color	Very blonde	Fair, not sparse, has a cowlick
Dysmorphic features	Low set ears, flat nasal bridge, strabismus in the left eye, thin lip, polydactyly	Thin upper lip; prominent lower lip; long smooth philtrum; small ears; brachycephaly.	Low set ears; short philtrum; thin upper lip; high palate; prominent chin
Peripheral spasticity	No	Yes	Yes
Truncal hypotonia	Yes	Yes	No
Developmental progress	Raised her head at 12 months but still unstably at 15months, still can't sit at age 15months	Smiled at 4 months; Sat supported −8 months; Rolling −9 months; not walking at 3	Sat at 15 months Walked with rollator—age 4; Can't walk unaided at age 14 years
Speech impairment	Makes some noises but no words.	Makes lots of noises but no words.	Single words at age 14 years
Visual defect	Strabismus and retinal detachment of left eye	Strabismus and hypermetropia,	None

**Figure 2:**
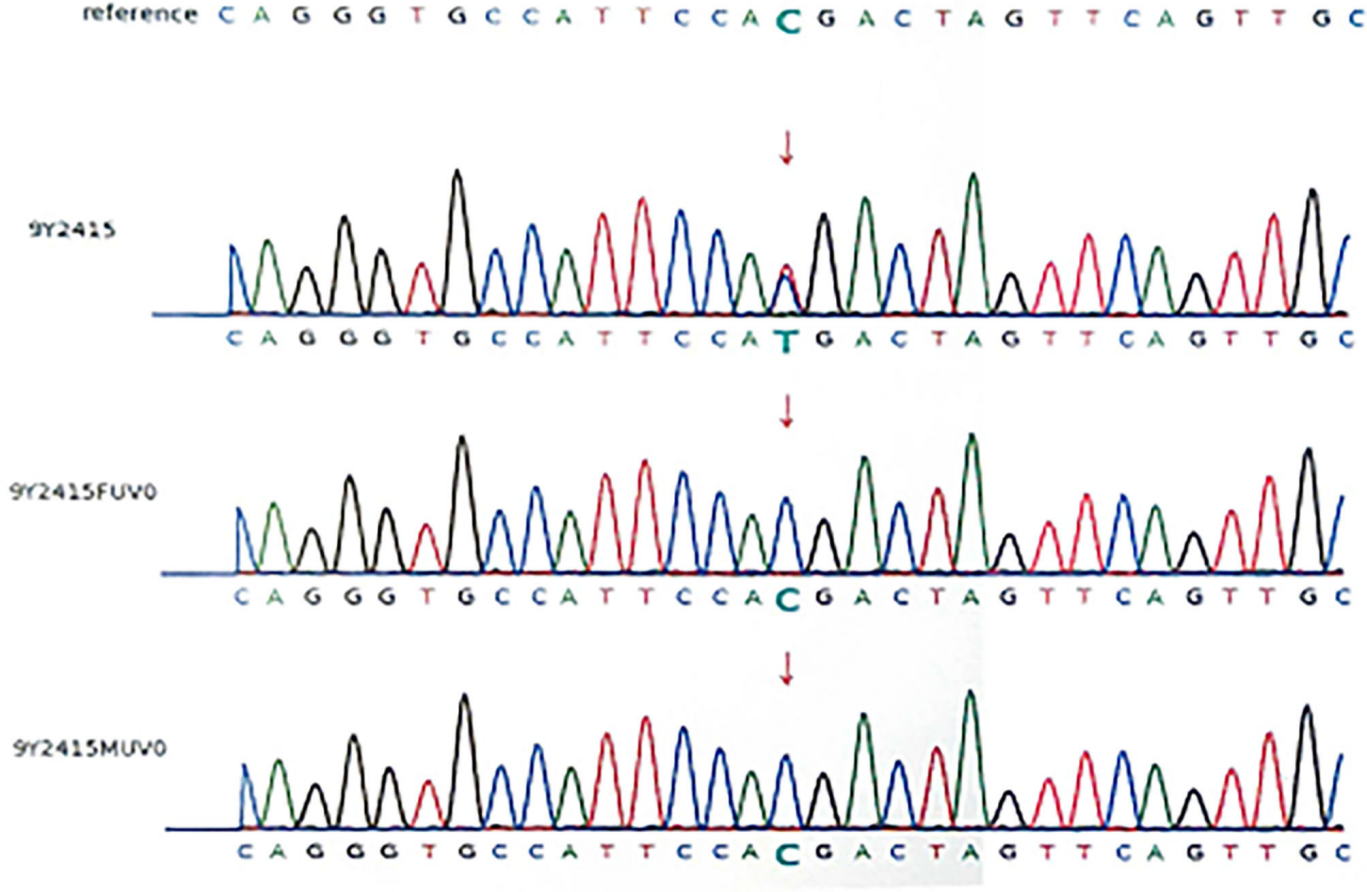
DNA electrophoregram with the c.1603C > T,p.R535X mutation in exon 10 of *CTNNB* 1. 9Y2415:patient; 9Y2415FUVO:father; 9Y2415MUVO:mother.

**Figure 3:**
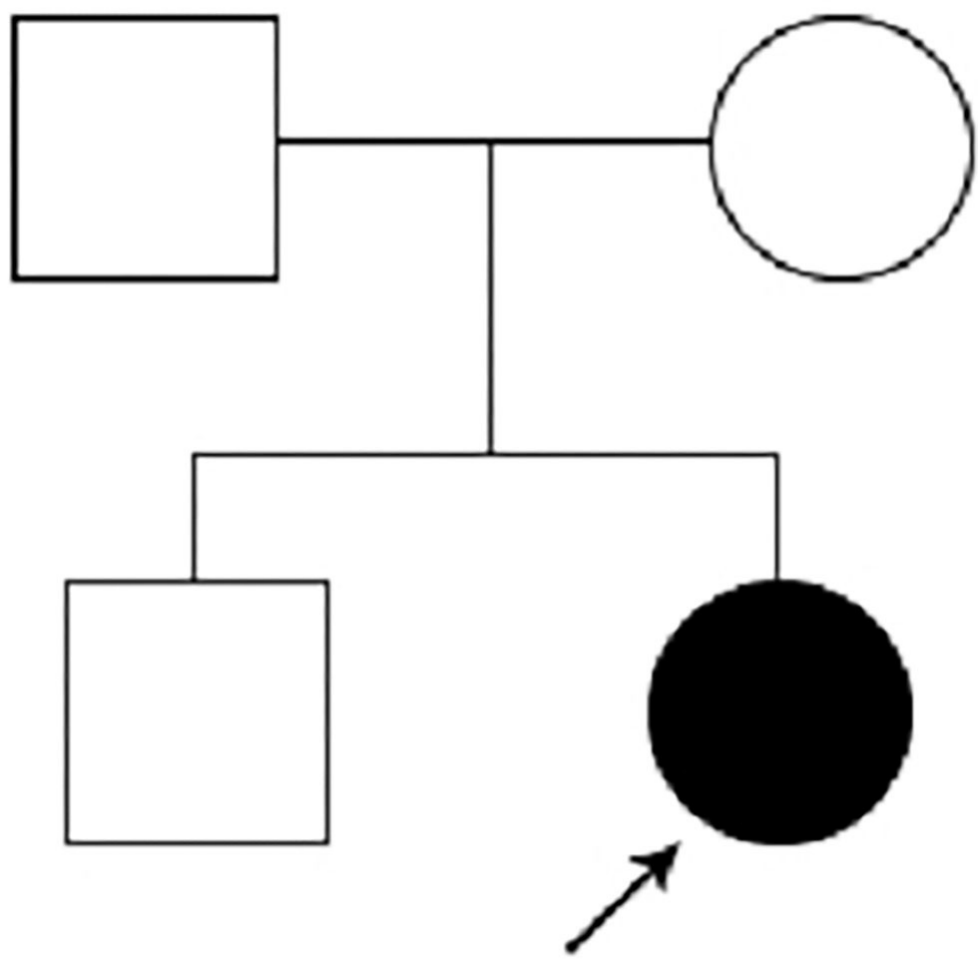
Pedigree of the family.

Because of her abnormal features (light hair color, fair skin, low ear position, flat nose, strabismus in the left eye, thin lips), microcephaly, hypotonia, development delay, retinal detachment, and polydactyly. Karyotype examination and the whole-exome sequencing were arranged for the patient, and her parents also did the whole-exome sequencing, because the patient's brother was healthy, he didn't take genetic test. Karyotype of the patients was normal, and a mutation c.1603C > T, p.R535X (gene position: CHR3: 41275708-41275708) was on exon 10 of chromosome 3, resulting in the loss of CTNNB1 gene function. The parents do not have the same gene mutation, then the child is identified as a *de novo* mutation (Figures 2, 3).

According to the clinical manifestations and gene mutation, the child was diagnosed as NEDSDV, and rehabilitation training was recommended. After 4 months of rehabilitation training, the child can raise her head steadily, but unable to sit, however, there was no significant improvement in language.

## Discussion

In this study, we present a 15-month-old Chinese girl with a complex phenotype, which included dysmorphic features (light hair color, fair skin, low set ears, flat nasal bridge, strabismus in the left eye, thin lip), microcephaly, hypotonia, development delay, retinal detachment, and polydactyly. WES of the patient revealed a *de novo* heterozygous nonsense mutation in exon 10 of the *CTNNB1* gene (c.1603C > T, p.R535X). This mutation had been reported in only two patients (2). All three patients had serious development delays, especially speech impairment. However, there were still some differences in the phenotype. The polydactyly and retinal detachment reported in our case has not been seen in the two cases previously reported.

*CTNNB1* gene is located on 3q22.1 and encodes β–Catenin protein. Its mutation is related to many diseases, such as a tumor, autism, and so on. In 2012, Joep de ligt et al. reported for the first time that three patients with intellectual disability had *CTNNB1* gene mutation, and their common phenotypes were: severe intellectual disability, absent or very limited speech, microcephaly, and spasticity with a severely impaired ability to walk. So far, 33 cases in the world have been reported. In 2017, the first case from Asian was reported (8), and as far as we knew, our case was the third case of the Asian population suffering from *CTNNB1* mutation related neurodevelopmental disorders.

The patient we reported was similar to the previous reports, but the polydactyly was not reported in the previous cases, and retinal detachment was also rare in the previous case reports, only two cases in the Asian population reported with retinal detachment (8, 10). However, the mutations of the *CTNNB1* were different from each other among the three patients. Previous studies have shown that heterozygous mutations in *CTNNB1* can cause non-syndromic familial exudative vitreoretinopathy(FEVR) and that FEVR was part of the *CTNNB1* haploinsufficiency phenotype (12), but the retinal detachment was only reported in the Asian population. Whether retinal detachment occurs in the Asian population is related to the genetic basis of the population, or because of the bias of too few reported cases, it needs to be further studied. Our patient expresses polydactyly, a unique symptom that has not been seen among other reported patients with *CTNNB1* mutation. Its occurrence may be related to the abnormal Wnt / β signal pathway caused by *CTNNB1* mutation. It has been found that mutation mice with polydactyly showed general de-regulated high levels of canonical Wnt/b-catenin signaling, it hypothesized that these Wnt/b-catenin signaling defects may contribute to the high proliferation rates in organ (13).

To date, 34 cases [one our case and 33 cases reported before (2, 5–11)] were reported. It appears to be a significant gender bias in those affected by inactivating *CTNNB1* mutations(22 females,12 males). Although this is a small sample from which to infer such a finding and that may become more obvious as more cases are identified. Thirty-three lost-function mutations of *CTNNB1* have been reported in the 34 patients, 19 nonsense mutations, nine frameshift mutations, three splice mutations, two complete gene deletions. The mutants were distributed in exons 3, 4, 5, 6, 7, 9, 10, 11, 12, 13, and introns 5, 7, and 10. Among the 34 patients, the mutations of 33 patients were *de novo*, while one patient inherited the mutation (c.734+1G>A) from her mother who had similar but slighter clinical manifestations (10). The mechanism of *CTNNB1* mutation leading to neurodevelopmental delay is not clear, but animal experiment identified that β-catenin loss could result in severe learning impairments, upregulation of γ-catenin (a partial functional homolog, whose neural-specific role is poorly defined) and reductions in synaptic adhesion and scaffold proteins which may affect brain development and function (14). Because of poor understanding of the molecular mechanisms of the disease, therapeutic strategies are limited. Most of the patients just were given rehabilitation training and symptomatic treatment. The progress is very poor, all patients suffered severe intellectual disability and can't live by themselves.

In conclusion, our study reported a heterozygous nonsense mutation in the *CTNNB1* gene (c.1603C > T, p.R535X) in a Chinese family with neurodevelopment disorder, retinal detachment, and polydactyly. As far as we know, this is the third case reported in the Asian population and with a special retinal detachment which was only be reported in the Asian population, and we also found that polydactyly may be a new feature of *CTNNB1* mutation. This report not only helps to expand the clinical phenotype spectrum of the *CTNNB1* mutation but also prompts a new insight into genetic diagnosis in patients with a neurodevelopmental disorder, retinal detachment, and polydactyly.

### Limitations and Strengths

We presented the clinical phenotype and genetic variation of a case with *de novo* CTNNB1 Nonsense Mutation, and firstly reported the phenotype of polydactyly in the CTNNB1 mutation. However, there were still some deficiencies, because of insufficient follow-up time, the development and prognosis of the case were unknown.

## Data Availability Statement

All datasets presented in this study are included in the article/ supplementary material.

## Ethics Statement

Written informed consent was obtained from the mother of the child for the publication of any potentially identifiable images or data included in this article.

## Author Contributions

ZK contributed to the acquisition, analysis of data, and writing the first draft. YC contributed to the conception of the work and revised the paper. All authors contributed to the article and approved the submitted version.

## Conflict of Interest

The authors declare that the research was conducted in the absence of any commercial or financial relationships that could be construed as a potential conflict of interest.
